# Similarity-driven compression during encoding supports biased but more precise working memory

**DOI:** 10.1167/jov.26.4.8

**Published:** 2026-04-09

**Authors:** Janna W. Wennberg, John T. Serences

**Affiliations:** 1 Department of Psychology, University of California, San Diego, La Jolla, CA, USA; 2 Department of Cognitive Science, University of California, San Diego, La Jolla, CA,USA; 3 Neuroscience Graduate Program, University of California, San Diego, La Jolla, CA, USA

**Keywords:** visual memory, working memory, inter-item competition

## Abstract

Visual working memory (VWM) allows us to maintain and manipulate information in service of behavioral goals. Navigating rich visual environments often involves holding multiple items in VWM—some of them very similar. Recent work suggests that inter-item similarity impairs memory precision during encoding but enhances precision during active memory maintenance. The present study tested whether this inter-item similarity benefit observed during memory maintenance was due to compressing similar items into summary representations. In Experiment 1, participants encoded sample displays with four colored circles into memory: two circles were similar to each other in color, two items were dissimilar to both each other and to the similar items. Using a retrospective cue (“retro-cue”) presented after the sample display, we manipulated the inter-item similarity of the remembered stimuli by cueing two similar items, one similar and one dissimilar item, or two dissimilar items. Consistent with prior work, we observed higher precision memory and attractive biases between similar items, consistent with compression. In Experiment 2 we observed a similarity benefit and attractive bias for similar items even in the absence of a retro-cue. Importantly, the magnitude of the similarity benefit and attractive bias was the same across valid and neutral cues, suggesting that similarity-based compression occurs relatively early in the trial, before the onset of the retro-cue. In Experiment 3, we manipulated the onset of the retro-cue to occur early during the delay period, and we replicated the results of Experiments 1 and 2. Together, these results suggest that inter-item similarity enhances VWM performance through compression that occurs early during the trial as opposed to during maintenance in memory.

## Introduction

You are helping a friend find their car in a crowded garage. While walking, you hold the image of your friend's car in mind, or in your visual working memory (VWM), to provide a temporary store for maintaining and manipulating information (e.g., the image of a car) to serve immediate behavioral goals (e.g., locating the car) (van Ede & Nobre, 2023).

Despite robust debate about the existence of capacity limits in VWM, there is strong consensus that increasing the number of items held in mind leads to reduced precision for each item (Luck & Vogel, 1997; Wilken & Ma, 2004). These performance decrements are assumed to stem from inter-item competition for finite resources, and researchers have long sought to disentangle the role of psychophysical similarity in inter-item interference. In the verbal domain, phonological similarity impairs performance (Coltheart, 1993; Conrad, 1964), and increasing semantic similarity increases interference—sometimes leading to false memories (Roediger & McDermott, 1995). In the domain of visual working memory some research suggests that—like verbal similarity—visual similarity impairs WM performance. For example, research on the “mixed category benefit” for storing complex objects in VWM suggests that more similar items are encoded in overlapping neural populations and that increasing overlap predicts increasing interference (Cohen, Konkle, Rhee, Nakayama, & Alvarez, 2014). This idea is analogous to more recent behavioral data suggesting that similar items are more confusable with psychophysically similar items than psychophysically dissimilar items, perhaps because of more overlap in population-level neural responses (Schurgin, Wixted, & Brady, 2020). However, other behavioral research has produced the opposite pattern of data, where similarity boosts memory performance, even with complex stimuli such as faces (Jiang, Lee, Asaad, & Remington, 2016; Lin & Luck, 2009). Importantly, and irrespective of whether similarity helped or hurt performance, these studies did not dissociate the impact of similarity on early sensory encoding vs. the maintenance of information in VWM. Thus it is possible that similarity has differing impacts on different processing stages as a function of low-level stimulus properties, potentially accounting for some prior contrary findings in the literature.

In addition to the perceived similarity of memoranda, other work suggests that the impact of similarity on VWM also critically depends on task demands. For instance, remembering highly similar items can cause memories to be repelled from each other when tasks require making fine discriminations between the memoranda. This repulsive distortion might be adaptive as it facilitates discrimination by biasing memories to exaggerate small differences between similar stimuli (Chunharas, Rademaker, Brady, & Serences, 2022; Lively, Robinson, & Benjamin, 2021; Scotti, Hong, Leber, & Golomb, 2021). Alternatively, people sometimes extract summary statistics of a display and exploit redundancies to store multiple similar items together in an ensemble representation to improve WM (Azarov, Grigorev, & Utochkin, 2024; Cowan & Chen, 2008; Norris & Kalm, 2021; Zhang & Du, 2022). This results in inter-item attraction and memory representations for individual items that are less biased toward the mean of the group, with an extreme case occurring when individuated items are replaced by a single integrated representation (Son, Oh, Kang, & Chong, 2020; Thalmann, Souza, & Oberauer, 2019). Even though these ensemble representations can result in a decrease in the accuracy of reporting individual items, combining representations can be optimal as it results in higher average memory precision across all grouped items (Brady, Konkle, & Alvarez, 2011; Brady & Alvarez, 2015; Corbett, 2017; Orhan, Sims, Jacobs, & Knill, 2014; Sims, Jacobs, & Knill, 2012; Utochkin & Brady, 2020). Importantly, the formation of ensembles can sometimes rely on long-term memory, with progressively higher-level representations referred to as “chunks” (e.g. Brady, Konkle, & Alvarez, 2009). However, when stimuli change unpredictably from trial to trial, as in much of the literature, hierarchical chunking is not likely to have a significant influence on processing and ensemble coding is more analogous to a general “compression” of information that results in attractive biases. Thus understanding the role of inter-item similarity in shaping representations in VWM requires considering more than the physical properties of the stimulus. Instead, encoding constraints and task demands also determine how the remembered information will be used to support goal directed behavior.

Given that both perceived similarity and task demands can shape how similarity impacts memory representations, we sought here to better understand how inter-item similarity influences compression during encoding versus during storage in memory. Addressing this question is important because prior work on compression and hierarchical chunking has primarily focused on the role of similarity in shaping overall memory performance, with less focus on understanding when these effects arise. Moreover, we were motivated by recent work that tangentially addressed the timing of compression using a continuous report procedure that compared memory for “homogeneous” displays of four colors or four orientations and “heterogeneous” displays with two colors and two orientations (Wennberg & Serences, 2024). Although not a direct manipulation of inter-item similarity per se, the homogeneous displays used by Wennberg and Serences (2024) had four items from the same feature space and thus a higher degree of inter-item similarity on average. They observed a robust benefit for heterogeneous displays that had lower average similarity across all four items, consistent with some prior work (Avital-Cohen & Gronau, 2021; Cai, Fulvio, Samaha, & Postle, 2022; Cohen et al., 2014). However, in a separate experiment they presented a retrospective cue (“retro-cue”) after the offset of the memory display that indicated which subset of the four encoded items had to be remembered over the delay period. The use of the retro-cue thus encouraged subjects to encode all four stimuli with equal priority and to better isolate the impact of maintaining two more similar items (e.g., two colors) from two more dissimilar items (e.g., one color and one orientation). Importantly, when using the retro-cue to isolate the effects of similarity during memory maintenance, there was better memory performance for homogeneous items compared to heterogeneous items. The authors concluded that inter-item similarity—in this case defined as encoding and remembering two items from the same feature space—increases competition during sensory encoding but that similarity does not have an impact once items are actively maintained in WM. That said, similarity was only defined at the level of category, and the two randomly chosen colors or orientations on a given trial were not necessarily “similar” in their respective features spaces on average.

**Figure 1.**
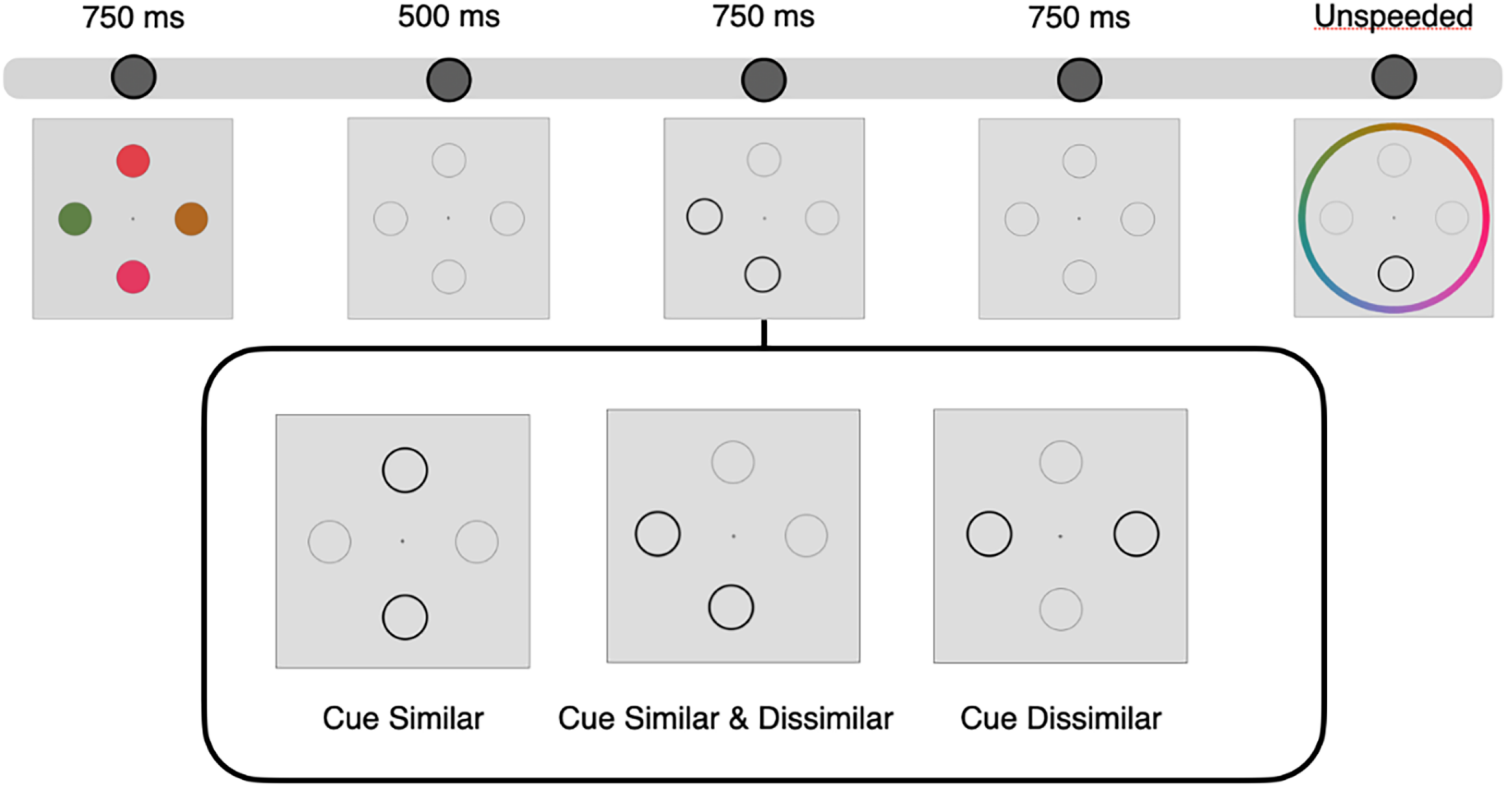
Procedure and conditions (Experiment 1). Participants were shown four items on each trial: two items were rotated 20° apart in color space (top and bottom item in this example), and the other two items (left and right item) were rotated 120° and −120° from the initial item.

Motivated by these findings, the present studies directly manipulated inter-item similarity within the same feature space to test the hypothesis that similarity-based compression primarily occurs during encoding as opposed to when information is actively stored in WM. We used a simple memory reproduction task where subjects had to click on the remembered feature from a continuous color wheel. Importantly, this task should generally encourage similarity-based compression, as that is theoretically optimal strategy to trade-off small attractive biases in individual representations for improved overall precision (Brady & Tenenbaum, 2013; Chunharas et al., 2022). In Experiment 1, participants remembered structured displays of colors where two colors were similar, and two colors were dissimilar to both each other and to the similar items. All four items were presented on each trial, and we retro-cued either the similar items or the dissimilar items and compared continuous report precision across retro-cue conditions (see Figure 1). Retro-cueing similar colors led to better memory performance compared to retro-cueing dissimilar colors, replicating some previous findings (Jiang et al., 2016; Lin & Luck, 2009), In addition, participants’ reports of similar items were attracted to each other, even when only one of the similar items was cued and relevant to the memory task. We hypothesized that the observed similarity benefit could be driven—in part—by compressing the similar items together early during the delay period, leading to both better memory and to magnified inter-item attractive biases. In Experiment 2, we retro-cued all four items on half of the trials (uninformative cue) and observed a precision benefit and attractive biases for similar items even in the absence of a retro-cue. Finally, in Experiment 3 we observed no change in precision or attractive biases even when an informative retro-cue was presented 100 ms after the sample display, suggesting that any compression had already occurred during encoding. The observation of attractive biases with a neutral cue indicates that similarity during encoding, even before cueing the relevant items to remember, can lead to better performance and to attractive biases.

### Open practices statement

All main and supplementary experiments were preregistered using a standard template from Open Science Framework (OSF) (Bowman et al., 2020). Preregistration plans, data, and code are available on Open Science Framework (OSF) (https://osf.io/mv5jt/).

Several experiments are not included in the main manuscript body for brevity, but results are available in a supplement in the same OSF repository linked above. Table 1 lists all experiments in chronological order.

**Table 1.** Chronological order of experiments. *Note*: All studies were preregistered.

Title	OSF title	*N*	In brief
S1	Retrocue-similarity-expS1	40	4-AFC task
S2a/2b	Retrocue-similarity-expS2a/S2b	50ea	2-AFC, manipulated targ-lure similarity
S3	Retrocue-similarity-expS3	50	2-AFC, targ-lure similarity, blocked design
Experiment 1	Retrocue-similarity-main-exp1	40	Continuous report
S4	Retrocue-similarity-expS4	40	Experiment 1 but with pre-cues
Experiment 2	Retrocue-similarity-main-exp2	40	Experiment 1 but manipulating retro-cue set size
Experiment 3	Retrocue-similarity-main-exp3	40	Experiment 1 but manipulating retro-cue onset

## Experiment 1

### Experiment 1 method

#### Participants

We recruited 40 participants using Prolific, and all participants completed the study online. Participants resided in the United States and were between the ages of 18–68 with a median age of 33. All reported normal or corrected-to-normal vision and no colorblindness. No participants who completed the study were excluded.

We performed an a priori power analysis using R (R Core Team, 2023) and simr (Green & MacLeod, 2016). Our effect of interest was whether retro-cueing dissimilar items impairs mnemonic precision, so we performed simulations using data from a previous study demonstrating a conceptually related effect with colorful circles and oriented bars (Wennberg & Serences, 2024). Using a cutoff of 90% power and an alpha-level of 0.05, we found that with 40 participants, we could detect an effect size 25% smaller than the one previously observed. This was a conservative estimate, because participants in the original study completed 60 trials per condition, whereas participants in the present study completed 100 trials per condition.

#### Stimuli

We used jsPsych, version 7, for stimulus presentation (de Leeuw & Gilbert, 2023). Participants were required to complete the experiment using a laptop or desktop computer, and they sat at an unknown distance from the monitor. Because this was an online study, monitors were not calibrated to render truly equiluminant colors. Although this may have produced a source of variation across participants, all experimental manipulations were within-subject.

Throughout the experiment, four placeholder circles and a central fixation cross were shown on a gray background. During each trial, four colorful circles appeared in each of the placeholder circles. We used a 360 circular color space matching Schurgin, Wixted, and Brady (2020) for all experiments. On all trials, the first color was selected uniformly from the space, and remaining colors were 20°, 120°, and −120° away from the first color, respectively. We intended to reverse the stimulus offsets so that half of trials had stimulus offsets 20°, 120°, and −120° from the first sampled item, and the other half had offsets of −20°, −120°, and 120°. However, because of a silent bug in the stimulus presentation software, all stimuli had offsets of 20°, 120°, and −120°. Colors were randomly assigned to spatial positions. Participants gave their response using a continuous color wheel, which randomly rotated from trial to trial to prevent the use of motor strategies throughout the experiment.

#### Procedure

Figure 1 shows a diagram of the experimental procedure. Participants clicked the central fixation cross to begin each trial. Four placeholder circles appeared on the screen throughout the duration of the experiment. After a 1000 ms delay, color stimuli appeared for 750 ms, followed by a 500 ms delay where the colors disappeared but the placeholder circles remained on the screen. Next, two items were retro-cued, and the placeholder circles of the cued items had thicker, darker borders for 750 ms. The retro-cues were 100% valid throughout the experiment. After the offset of the retro-cues, there was another 500 ms delay before the report period. During the report period, one item was probed for report (this item was always one of the retro-cued items). The placeholder circle of the probed item had a darker border, and participants had unlimited time to click the location on the color wheel that best matched the color shown in the probed location. Participants completed a total of 300 trials, and although there were no structured blocks, participants could take breaks between trials as needed.

The main experimental manipulation was changing the items that were retro-cued. In the Cue Similar condition, we retro-cued two similar items (the original item and the item 20° away). In the Cue Similar & Dissimilar condition, we cued the original item and either the item 120° or −120° away. In the Cue Dissimilar condition, we cued the items 120° and −120° away from the original item. We included this Cue Dissimilar condition so that participants did not learn that at least one of the two similar items is always cued and probed for report.

#### Data analysis

Data from each participant was uploaded to a secure server as a text file. We performed analyses using R. To combine, clean, and aggregate our data, we used the *tidyverse*, *plyr,* and *jsonlite* packages (Ooms, 2014; Wickham, 2020; Wickham, 2023). We used the *circular*, *lme4, lmerTest*, and *emmeans* packages to perform our analyses, and *ggplot2* and *viridis* for data visualizations (Agostinelli & Lund, 2023; Bates, Machler, Bolker, & Walker, 2014; Garnier, 2023; Garnier et al., 2023; Kuznetsova, Brockhoff, & Christensen, 2017; Lenth, 2025; Wickham, 2016). Before our main analyses, we cleaned the data by removing practice trials and changing each participant's unique Prolific ID to a standard participant ID for the protection of anonymity.

To assess whether mnemonic precision differed across retro-cue conditions, we computed the circular standard deviation of the continuous error distribution for each participant and each condition. We fit linear mixed effects models with circular standard deviation as the response variable and retro-cue condition as a fixed effect. Participants were entered as random effects. We used linear mixed effects models rather than repeated measures ANOVAs because linear mixed effects models are more flexible and robust to violations of normality (Knief & Forstmeier, 2021). We examined the Type III ANOVA output of the model to test for an effect of retro-cue condition and used a significance threshold of *p* < 0.05. Degrees of freedom were calculated using Kenward-Roger approximation. We preregistered that we would perform follow-up planned comparisons to compare each retro-cue condition separately, regardless of the result of the ANOVA. We followed Chunharas et al. (2022) to assess whether response distributions were biased toward or away from distractors, as well as whether the degree of bias differed reliably across retro-cue conditions.

### Experiment 1 results

#### Mnemonic precision

**Figure 2.**
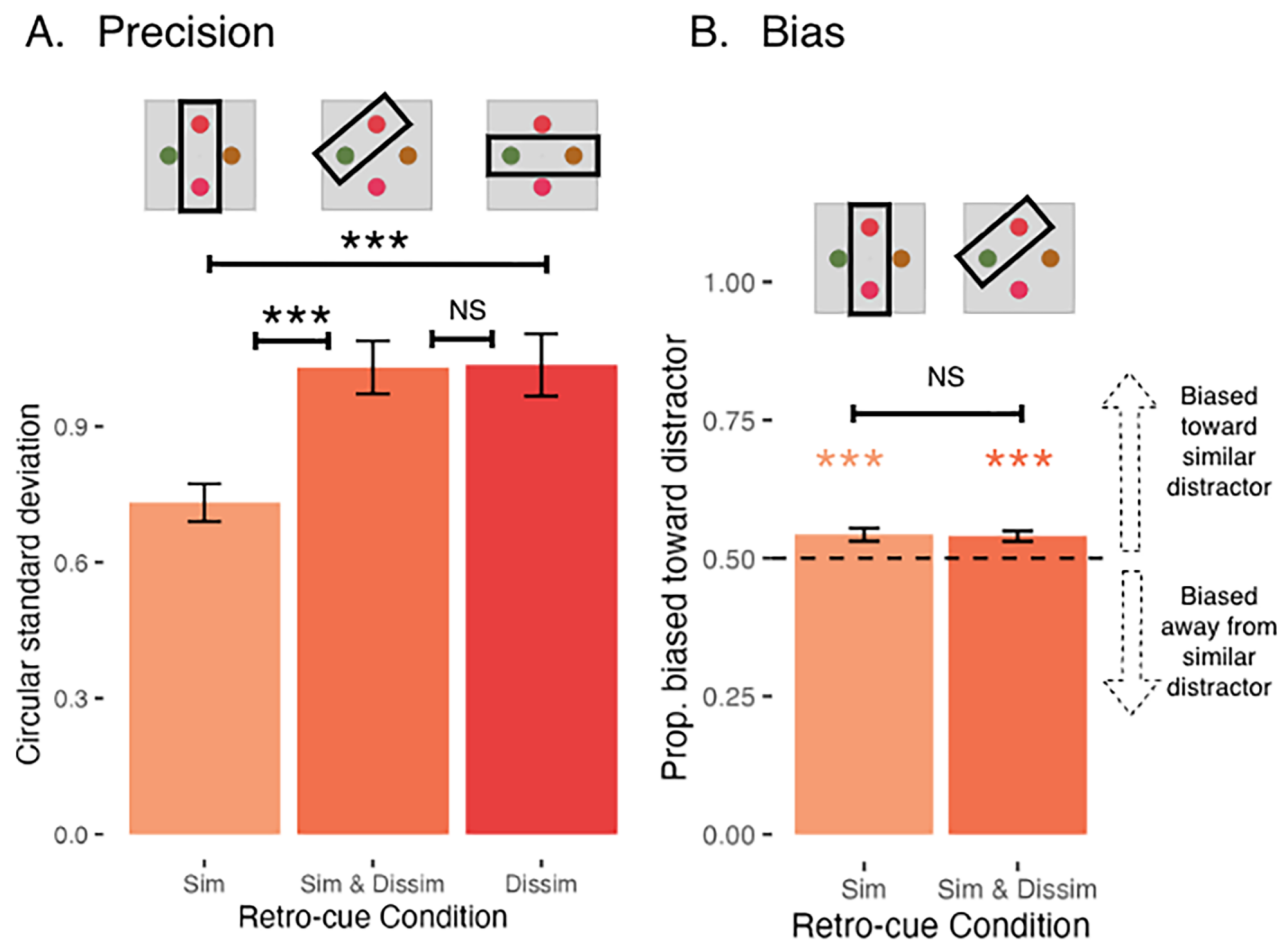
Mean precision and bias (Experiment 1). (A) Mean continuous report precision by retro-cue condition, measured by the circular standard deviation of the response distribution (in radians). Asterisks indicate significant pairwise differences (*p < 0.05, **p < 0.01, ***p < 0.001). (B) Mean bias by retro-cue condition (omitting Cue Dissimilar), measured by the proportion of trials rotated toward the similar distractor. Asterisks indicate that the proportion of trials rotated towards the mean is reliably above 0.50 (*p < 0.05, **p < 0.01, ***p < 0.001). Error bars represent the standard error of the mean.

A plot of the mean circular standard deviation is shown in Figure 2A. Participants were reliably the most precise in the Cue Similar condition. There was a main effect of retro-cue condition (*F*(2, 78) = 30.07, *p* = 2.09 × 10^−10^), and follow-up planned comparisons showed that participants were more precise in the Cue Similar condition than the Cue Similar & Dissimilar condition as well as the Cue Dissimilar condition (all *t*(78) < −6.65, all *p* < 0.0001). There was no evidence for a reliable difference between the Cue Similar & Dissimilar and the Cue Dissimilar conditions (*t*(78) = −0.12, *p* = 0.99). A plot of the mean mnemonic precision in each retro-cue condition is shown in Figure 2.

#### Bias

Participants showed an attractive bias in both the Cue Similar and Cue Similar & Dissimilar conditions. The proportion of trials rotated towards the similar distractor (i.e., the proportion of trials with attractive bias) was reliably above 0.5 in both retro-cue conditions [Cue Similar: *t*(72.4) = 4.00, *p* = 0.0001; Cue Similar & Dissimilar: *t*(72.4) = 3.74, *p* = 0.0002]. There was no significant main effect of retro-cue condition [*F*(1, 39) = 0.05, *p* = 0.83], suggesting that the two conditions did not vary significantly in the proportion of trials with attractive bias.

### Interim discussion

In Experiment 1, memory recall was reliably more precise when two similar items were retro-cued than when two dissimilar items were retro-cued. This pattern of data is conceptually consistent with Wennberg and Serences (2024), where participants were more accurate when two colors were retro-cued than when one color and one orientation were retro-cued. Notably, we observed reliable attractive biases between the similar items: participants’ reports of similar items were rotated towards the similar item, even when that item was not retro-cued and was thus task irrelevant.

After data collection and analysis, we hypothesized that—based on the observation of attractive biases toward non-cued similar items—participants may be compressing the similar items together during sensory encoding, giving rise to a bias that persists during the memory delay period. In Experiment 2, we added a neutral retro-cue on half of the trials such that all four items were cued, and thus no information was provided about which stimulus was going to be probed during the memory recall phase. This enabled us to assess attraction between similar items even when subjects didn't know what items to prioritize during the delay period.

If compression was primarily happening—and potentially increasing in magnitude—during the later parts of the memory delay period, then we reasoned that attractive biases should be larger when two similar items are informatively retro-cued compared to when all four items are neutrally cued (i.e., uninformatively cued). This is because a retro-cue manipulation equates the encoding period while independently varying memory demands (i.e. remembering only the two similar items or remembering all four items). However, if compression happens before the onset of any retro-cue, the magnitude of attractive biases should be the same across informative and uninformative retro-cues because all compression will be complete by the time the retro-cue is presented and subjects can either sub-select only the relevant stimuli or maintain a representation of all four items.

## Experiment 2

### Experiment 2 method

#### Participants

We analyzed data from 40 participants from Prolific using the same inclusion criteria as Experiment 1. Participant ages ranged from 19–67 years with a median age of 38 years. Participants completed half as many trials per condition as they did in Experiment 1. However, this choice was motivated by a re-analysis of Experiment 1 revealing reliable effects using only half of the total trials. A total of 44 participants completed the experiment, but data files from four participants were not uploaded to the server due to a network communication error. No participants were excluded for performance reasons.

#### Stimuli

Stimuli were nearly identical to Experiment 1 except that on half of the trials we retro-cued all four displayed items so that subjects had no advance information regarding which stimulus would be the memory probe (termed an “uninformative cue”). We chose to cue all items rather than leave the screen blank to control for any masking effect of the retro-cue stimuli that might occur when retro-cueing two items. Remaining trials where two items were retro-cued (as in Experiment 1) were termed “informative cue trials.”

We also fixed several issues to improve the study design. First, we fixed the silent bug in Experiment 1 that led to only 0°, 20°, 120°, and −120° items being presented (and not 0°, −20°, −120°, and 120° items). Although we do not believe this bug significantly altered our results, we ensured that stimuli were presented as intended for the present experiment. Finally, in Experiment 1, participants were always probed to the 0° item in the Cue Similar Condition. In Experiment 2, we probed the 20° item on half of Cue Similar trials.

#### Procedure

**Figure 3.**
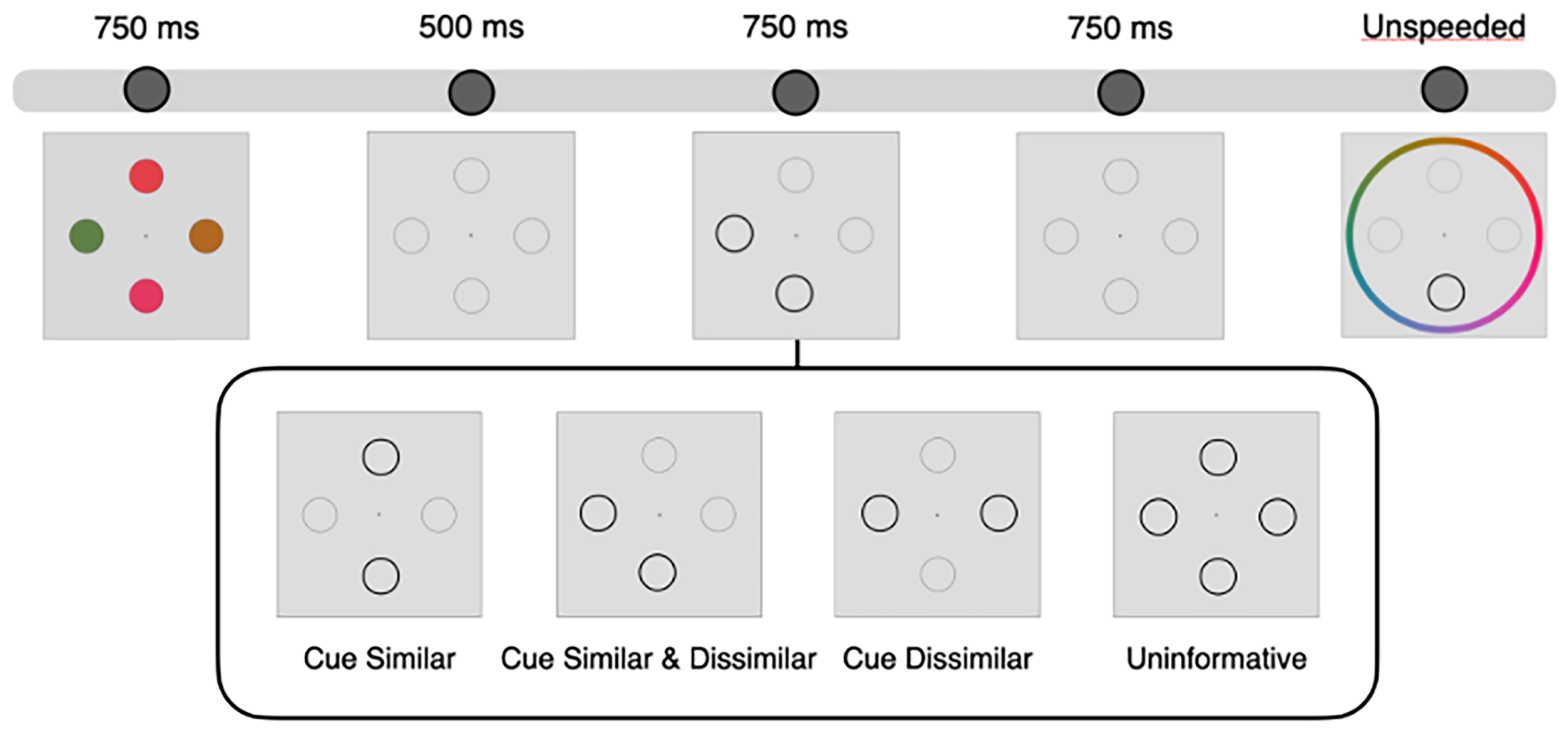
Procedure and conditions (Experiment 2). Stimuli were the same as Experiment 1. Two items were retro-cued on half of trials, and four items were cued on the other half of trials.

A diagram of the task is shown in Figure 3. The procedure was identical to Experiment 1, with the exception that we incorporated an uninformative cue that highlighted all four items on half of trials.

#### Data analysis

Data aggregation and cleaning mirror Experiment 1. To directly replicate Experiment 1, we first subsetted our data to include retro-cue 2 trials only and performed the same analyses that we did in Experiment 1. Note that when the retro-cue was uninformative (i.e., all four items were cued), we could not perform all analyses to perfectly mirror those in Experiment 1 because Experiment 1 compared performance when two similar items and two dissimilar items were retro-cued. Thus, on uninformative-cue trials, instead of comparing precision and bias across retro-cue conditions, we compared precision and bias across probed items (i.e., trials where a similar item was probed and trials where a dissimilar item was probed).

### Experiment 2 results

#### Replicating Experiment 1

To assess whether Experiment 1 results replicated, we first analyzed only informative retro-cue trials (i.e., when two items were retro-cued).

##### Mnemonic precision

**Figure 4.**
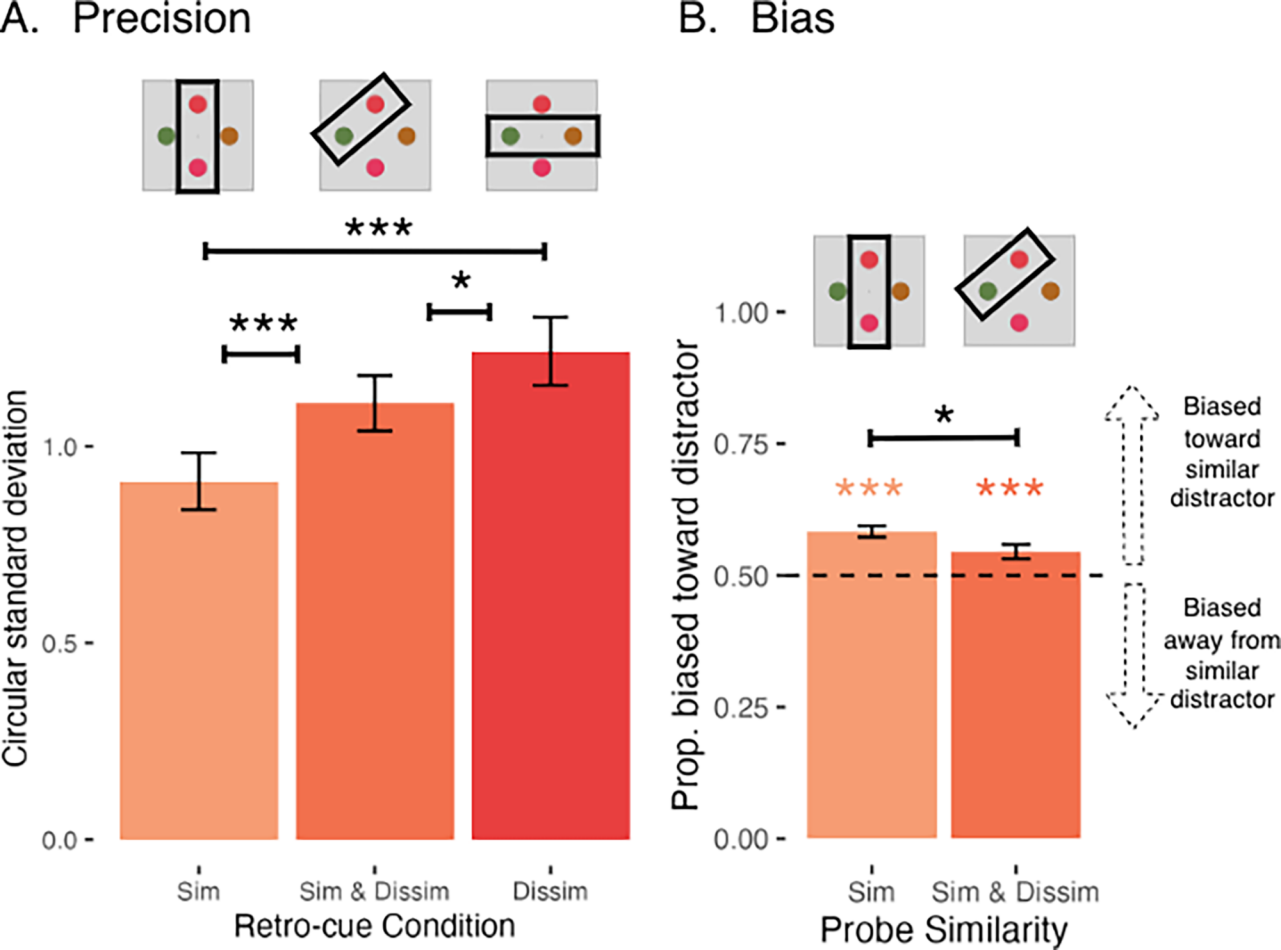
Mean precision and bias (Experiment 2—informative retro-cues only). (A) Mean continuous report precision by retro-cue condition, measured by the circular standard deviation of the response distribution (in radians). Asterisks indicate significant pairwise differences (*p < 0.05, **p < 0.01, ***p < 0.001). (B) Mean bias by retro-cue condition, measured by the proportion of trials rotated toward the mean of the similar colors. Asterisks indicate that the proportion of trials rotated towards the mean is reliably above 0.50 (*p < 0.05, **p < 0.01, ***p < 0.001). Error bars represent the standard error of the mean.

The mean precision in each retro-cue condition is shown in Figure 4A. When two items were retro-cued, precision worsened in a stepwise manner across retro-cue conditions: participants were the most precise in the Cue Similar Condition, followed by Cue Similar & Dissimilar, followed by Cue Dissimilar. The differences between each pair of conditions were significant [Cue Similar vs. Cue Similar & Dissimilar: *t*(78) = −4.30, *p =* 0.0001; Cue Similar vs. Cue Dissimilar: *t*(78) = −7.16, *p* < 0.0001; Cue Similar & Dissimilar vs. Cue Dissimilar: *t*(78) = −2.86, *p* = 0.015]. There was a significant main effect of retro-cue condition [*F*(2, 78) = 26.01, *p* = 2.22 × 10^−9^].

##### Bias

 Figure 4B shows the mean proportion of trials with attractive bias in the Cue Similar and Cue Similar & Dissimilar conditions. When analyzing trials with two items cued (informative retro-cues), responses on Cue Similar trials and Cue Similar & Dissimilar trials were reliably rotated toward the similar distractor [Cue Similar: *t*(76.7) = 6.72, *p* < 0.0001; Cue Similar & Dissimilar: *t*(76.7) = 3.65, *p* = 0.0002]. There was a reliable main effect of retro-cue condition [*F*(1, 39) = 5.41, *p* = 0.03], and participants showed more attractive bias in the Cue Similar condition than the Cue Similar & Dissimilar condition.

#### Comparing across informative and uninformative retro-cues

Because retro-cue condition was not defined with neutral cues, we used probe similarity as our primary independent variable when comparing across informative and uninformative retro-cues. Probe similarity refers to whether the probed item was one of the similar or dissimilar memoranda.

##### Mnemonic precision

**Figure 5.**
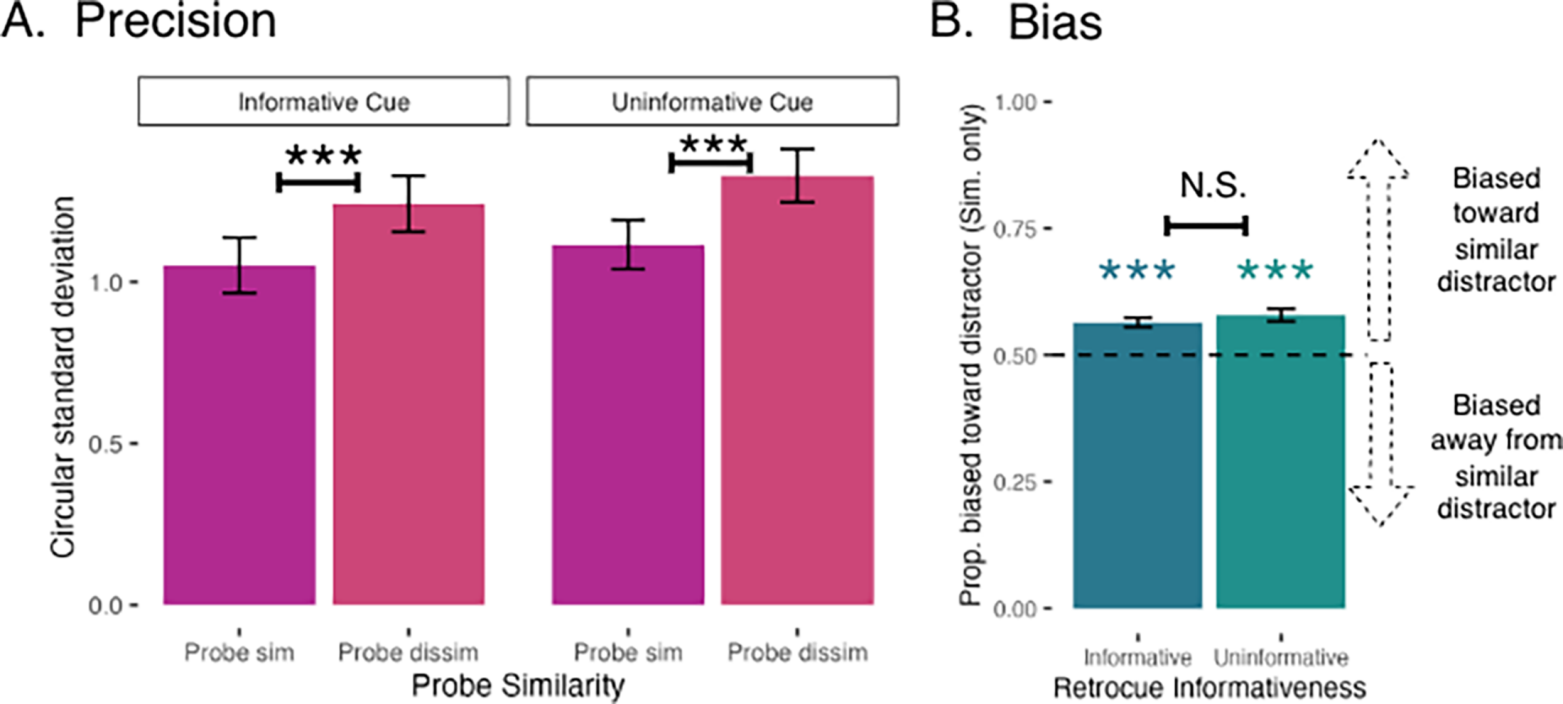
Mean precision and bias (Experiment 2—informative and uninformative retro-cues). (A) Mean continuous report precision by retro-cue informativity and probe similarity, measured by the circular standard deviation of the response distribution (in radians). Asterisks indicate significant pairwise differences (*p < 0.05, **p < 0.01, ***p < 0.001). (B) Mean bias by retro-cue informativeness (Probe Sim trials only), measured by the proportion of trials rotated toward the similar distractor. Asterisks indicate that the proportion of trials rotated toward the mean is reliably above 0.50 (*p < 0.05, **p < 0.01, ***p < 0.001). Error bars represent the standard error of the mean.

The mean precision for each condition is shown in Figure 5A. Critically, there was a main effect of retro-cue information such that participants were more precise for informative retro-cues (i.e., when two items were retro-cued) than for neutral, uninformative cues [*F*(1, 117) = 4.76, *p* = 0.031]. This is in line with a successful retro-cue manipulation and indicates that participants were not trying to remember all four items on trials with an informative retro-cue.

Although the full model including an interaction term did not yield a significant main effect of probe similarity [*F*(1, 117) = 2.28, *p* = 0.133] or an interaction [*F*(1, 117) = 0.12, *p* = 0.73], an additive model excluding the interaction revealed a robust main effect of probe similarity [*F*(1, 118) = 33.99, *p* = 4.92 × 10^−8^], suggesting a consistent influence of probe similarity across levels of retro-cue information. Follow-up analyses using estimated marginal means further supported this, revealing a significant effect of probe similarity at each level of retro-cue information [all *t*(118) > 5.8, all *ps* < 0.0001]. The model without an interaction term yielded a main effect of retro-cue set size as well [*F*(1, 118) = 4.80, *p* = 0.03].

##### Bias

 Figure 5B shows the mean proportion of trials with attractive bias in informative and uninformative retro-cue conditions—because bias was uninterpretable when dissimilar items were probed, here we show only trials where a similar item was probed. Participant responses were reliably biased toward the similar distractor when the cue was informative [*t*(60.5) = 5.82, *p* < 0.0001] and uninformative [*t*(60.5) = 7.15, *p* < 0.0001]. There was no significant main effect of retro-cue informativity [*F*(1, 39) = 1.91, *p* = 0.17], suggesting that the magnitude of bias was not reliably different between these conditions.

### Interim discussion

In Experiment 2, we observed a reliable similarity benefit, where items were reported more accurately when they were similar to other items than when they were dissimilar. This replicates the main observation from Experiment 1 using an independent sample ensuring that our initial finding is robust and that changes implemented during Experiment 2 did not alter the conclusions. We also replicated the finding that reports of similar items were attracted towards each other, and we observed a strong similarity benefit even with uninformative retro-cues, suggesting that similar items are compressed together early during the trial. The main effect of retro-cue information suggests that participants were using the cues as intended and not holding onto all four items throughout the delay period. We observed similar compliance in prior work using a similar paradigm as well (Wennberg & Serences, 2024). Finally, the magnitude of the attractive biases did not differ across the informative and uninformative cues, suggesting that most compression was done by the onset of the cue. In Experiment 3, we manipulated the onset of the retro-cue to occur even earlier in the delay period on half of trials. This provided an opportunity to replicate the general findings reported above and to directly assess if grouping is finished shortly after stimulus offset.

## Experiment 3

### Experiment 3 method

#### Participants

Using the same sample size justification and inclusion criteria as Experiment 2, we analyzed data from 40 participants. Recruitment procedures, informed consent, and compensation were the same as prior experiments. A total of 43 participants completed the task, but data from three participants did not upload to the server. Participants were 19–69 years of age with a median age of 37 years of age.

#### Stimuli

Stimuli for Experiment 3 were identical to Experiments 1 and 2.

#### Procedure

On all trials, color stimuli and retro-cues were presented for 750 ms each, mirroring Experiments 1 and 2. The critical difference from Experiment 1 is that we manipulated the lengths of the blank delay periods before and after retro-cue presentation, but we kept the total length of the delay period constant. On trials with an early cue onset, there was a 100 ms blank delay before the onset of the retro-cues and a 1150 ms delay after retro-cue offset. On trials with a late cue onset, there was a 750 ms blank delay before retro-cue onset and a 500 ms blank delay after retro-cue offset.

#### Data analysis

Data analysis procedures followed Experiments 1 and 2.

### Experiment 3 results

#### Mnemonic precision

**Figure 6.**
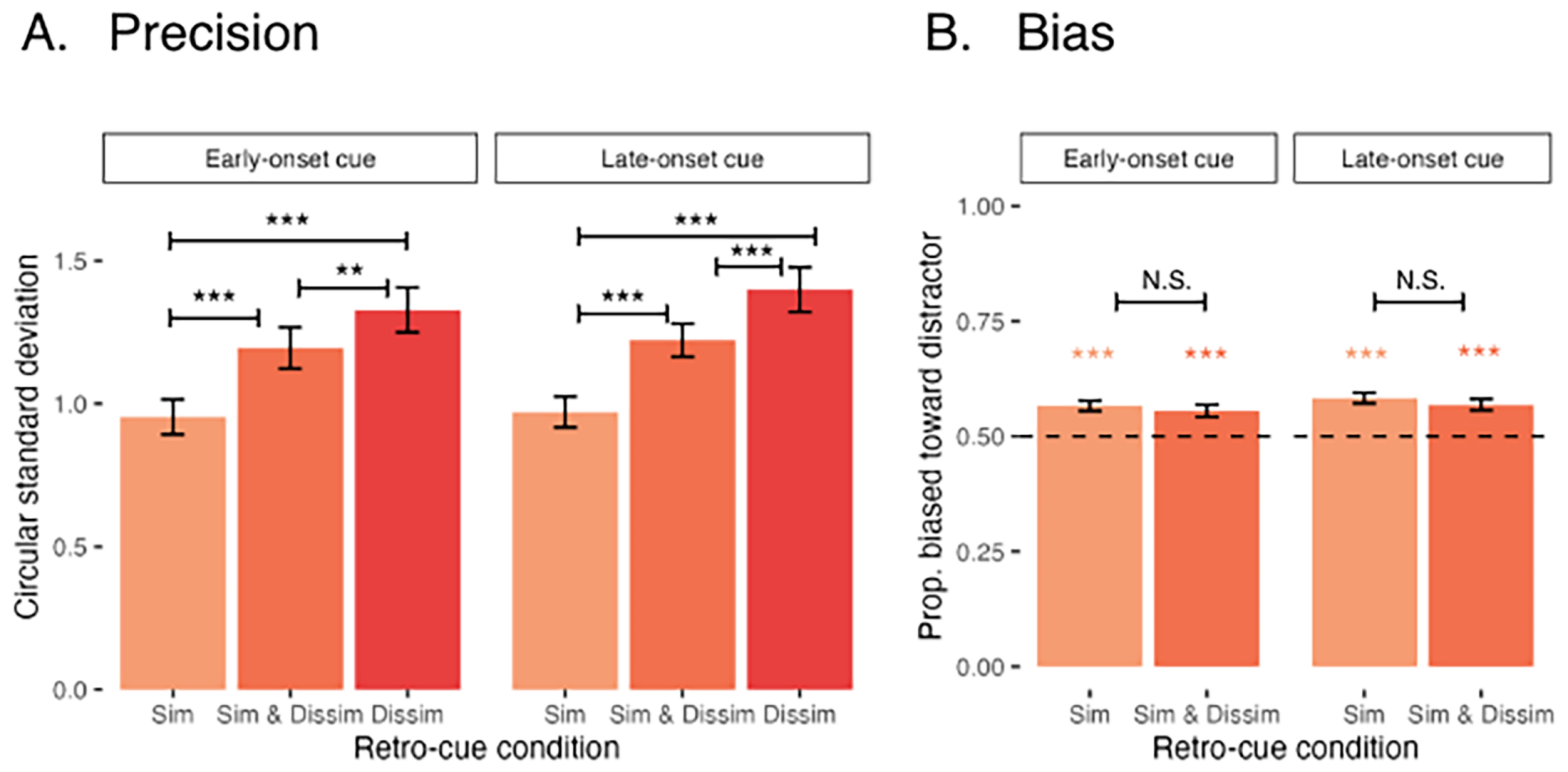
Mean precision and bias (Experiment 3). (A) Mean continuous report precision by retro-cue onset time and retro-cue condition, measured by the circular standard deviation of the response distribution (in radians). Asterisks indicate significant pairwise differences (*p < 0.05, **p < 0.01, ***p < 0.001). (B) Mean bias by retro-cue onset time and retro-cue condition (omitting Cue Dissimilar trials), measured by the proportion of trials rotated toward the distractor. Asterisks indicate that the proportion of trials rotated towards the mean is reliably above 0.5 (*p < 0.05, **p < 0.01, ***p < 0.001). Error bars represent the standard error of the mean.

A plot of the mean mnemonic precision in each retro-cue condition and each display-cue SOA is shown in Figure 6A. There was a significant effect of retro-cue condition [*F*(2, 195) = 82.04, *p* = 2.00 × 10^−16^]. In both cue onset conditions, mnemonic precision was best in the Cue Similar Condition, followed by Cue Similar & Dissimilar, followed by Cue Dissimilar, and the difference in precision between each pair of conditions was significant [all *t*(195) < −2.98, all *p* < 0.009]. There was no significant effect of cue onset time [*F*(1, 195) = 2.21, *p* = 0.14], and no significant interaction between retro-cue condition and cue onset time [*F*(2, 195) = 0.41, *p* = 0.67].

#### Bias

A plot of the proportion of trials with attractive bias in each condition is shown in Figure 6B. At all levels of retro-cue condition and cue onset time, responses were reliably biased toward the similar distractor [all *t*(149) > 4.54, all *ps* < 0.0001]. There were no significant effects of retro-cue condition [*F*(1, 117) = 1.16, *p* = 0.28] or cue onset time [*F*(1, 117) = 1.77, *p* = 0.17], nor was there a significant interaction [*F*(1, 117) = 0.02, *p* = 0.88]. There was no reliable difference in attractive bias between Cue Similar and Cue Similar & Dissimilar conditions for either early cue onset trials [*t*(117) = 0.66, *p* = 0.52] or late cue onset trials [*t*(117) = 0.87, *p* = 0.39].

## General discussion

Across three online behavioral experiments, we provide evidence that similar items are compressed together early during the trial, which improves WM precision. In Experiment 1, participants viewed displays with two similar and two dissimilar colors and were retro-cued to recall either similar or dissimilar items. Retro-cueing similar items improved memory precision and led to reports biased towards the similar distractor, even when the similar distractor was not cued—suggesting early compression of similar items that may enhance memory and cause inter-item attraction. Experiment 2 replicated the similarity benefit even with uninformative retro-cues, demonstrating that similarity-based compression is not dependent on the selective maintenance of information in WM. In Experiment 3, we varied the delay between display offset and retro-cue onset to test whether similarity-based compression occurred early during the delay period. We further replicated the similarity benefit and observed a similar bias magnitude with early and late retro-cues, suggesting that trials with early-onset and late-onset cues have the same amount of data compression, lending support to the hypothesis that similarity-based compression occurs before the relevant items are cued for selective storage. In all three experiments, we observed that responses were biased away from the mean of the similar items when two dissimilar items were retro-cued, resulting in an apparent repulsion effect. We believe that this is an artifact of how our stimuli were selected and not indicative of a bias in memory representations: due to the relative angles of the stimuli, reporting dissimilar items without bias will result in an apparent repulsion because the reports will be—on average—rotated away from the mean of the similar items.

The present work suggests that even before cue onset, task demands shape memory representations to support higher precision memory recall. Because WM representations of individual items contain ensemble-like properties, WM information is stored at multiple levels of abstraction, leading to attractive distortions of individual items towards their mean (Brady & Alvarez, 2011; Corbett, 2017; Utochkin & Brady, 2020). While attractive distortions may alter memory representations, the increased stability of an ensemble representation far outweighs the cost, making this compression an optimal strategy (Brady & Tenenbaum, 2013). The present study replicated prior work showing that similar items may be remembered more precisely than dissimilar items because similar items are compressed together (Allen, Brady, & DeStefano, 2021; Brady & Alvarez, 2015; Lively et al., 2021; Son et al., 2020). Furthermore, these adaptive distortions occur early during the trial, supporting the view that grouping shapes early stimulus-evoked representations to support higher-precision WM representations.

Based on our prior work (Wennberg & Serences, 2024), we hypothesized that inter-item similarity impairs WM performance early during the trial but enhances WM during maintenance, after the relevant items have been cued. Instead, we found here that inter-item similarity enhanced performance early on—a result consistent with previous studies finding that inter-item similarity enhanced performance more generally (Jiang et al., 2016; Lin & Luck, 2009) but that might be inconsistent with our earlier work (Wennberg & Serences, 2024). We propose two explanations that are not mutually exclusive. First, Wennberg and Serences (2024) compared displays of items from the same feature space (e.g. two colors) to displays of items from different feature spaces (e.g., one color and one orientation). Thus similar items merely belonged to the same feature space and similarity within a feature space was not manipulated. As a result, the pairwise average of the colors was not as similar as the similar colors in the present study. Second, displays with two colors and two orientations may have resulted in items being compressed along feature dimensions such that participants grouped the colors together and the orientations together. This may have facilitated performance for displays where participants remember two groups of items from different feature categories compared to displays where participants remember four items from the same feature category (and the four items from the same feature category may not have been very similar to one another).

Despite clear evidence for similarity-based compression early in the trial (i.e., within 100 ms after sample offset), it is possible that our findings do not generalize to all stimuli. For example, Diaz, Vogel, and Awh (2021) show electrophysiological evidence that grouping items based on collinearity may occur later in visual processing (e.g., around 500 ms after stimulus offset). It is possible that grouping items based on psychophysical similarity involves forming a gist representation where items are remembered relative to one another, but items are not integrated into a single object, per se. Thus multiple processes that fall under the putative terms of “compression” or “chunking” may recruit different cognitive and neural processes that influence their respective timecourses.

In sum, our results provide evidence that similar items are compressed together early during the delay and that this compression is finished prior to any cue-based selection. Moreover, similarity introduces distortions, but nevertheless improves overall recall precision. The improved performance is likely due to grouping being an optimal strategy under many scenarios, as it leads to more stable representations compared to holding individuated representations (Brady & Tenenbaum, 2013; Chunharas et al., 2022; Utochkin & Brady, 2020). By revealing that similarity-based compression occurs early, our findings underscore the prominent role of early sensory processing in producing biases and influencing the overall precision of working memory representations.
